# Characterization of Angle Accuracy and Precision of 3-Degree-of-Freedom Absolute Encoder Based on NanoGPS OxyO Technology

**DOI:** 10.3390/s20123462

**Published:** 2020-06-19

**Authors:** Marco Pisani, Milena Astrua, Pierre-Alix Carles, Stefan Kubsky, Thanh-Liêm Nguyên, Olivier Acher

**Affiliations:** 1 Division of Applied Metrology and Engineering, INRiM, National Institute for Metrological Research, strada delle Cacce 91, 10135 Torino, Italy; m.pisani@inrim.it (M.P.); m.astrua@inrim.it (M.A.); 2 IPVF, Institut Photovoltaïque d’Île-de-France, 18 Boulevard Thomas Gobert, 91120 Palaiseau, France; pierrealix.carles@gmail.com; 3 Synchrotron SOLEIL, L’Orme des Merisiers, 91192 Gif-sur-Yvette, France; stefan.kubsky@synchrotron-soleil.fr; 4 HORIBA France, 14 Boulevard Thomas Gobert, 91120 Palaiseau, France; thanh-liem.nguyen@horiba.com

**Keywords:** vision-based sensor, encoder, angle measurement, metrology, position control.

## Abstract

An absolute encoder based on vision system nanoGPS OxyO was developed by HORIBA France. This encoder provides three types of position information, namely, two inplane co-ordinates and inplane angular orientation. This paper focuses on the characterization of its angular performance. To this aim, the nanoGPS OxyO system was compared with the national angle standard of the National Metrology Institute of Italy (INRIM) that had evaluated accuracy of about 0.1 µrad. The effect of image size and illumination conditions on angular measurements was studied. Precision better than 10 µrad and accuracy better than 63 µrad over 2π rotation were demonstrated. Moreover, the application of nanoGPS OxyO to the characterization of rotation bearing is presented. Small deviations from pure rotational behavior were evidenced that would have not been possible using laser interferometers. As a consequence of its accuracy and versatility, the nanoGPS OxyO encoder is expected to be useful for laboratory experiments and quality-control tasks.

## 1. Introduction

Position encoders are sensors that provide information on the relative position of a part of a mechanical system with respect to another part, and they are key components of numerous scientific instruments, automation systems, and robots, along with a variety of automated applications. Two main types of encoders are available: incremental (that determine a co-ordinate change with respect to an arbitrary position) and absolute encoders (that provide absolute co-ordinate information). Position encoders were successfully developd from a large variety of physical approaches, including magnetics, electrics, and optics [1,2], leading to a large variety of encoders that meet different needs in terms of precision, accuracy, range, speed, integration constraints, cost, etc.

While a number of encoders provide information on a single co-ordinate (i.e., linear or angular encoders), some can provide several co-ordinates in a single reading [2]. This is the case of position encoders that are based on vision systems. These encoders record the image of specially engineered scales and determine, through software treatment, linear co-ordinates x, y and orientation φ_z_ of the scale [3,4,5,6,7,8,9,10,11,12,13]. In vision-based encoders, a significant variety of base patterns have been used to construct coded scales, and a significant variety of imaging systems have been used to read the scales, ranging from high-quality microscopes to cheap cellphone cameras. While the general principle of vision-based 3-degree-of-freedom (3-DOF) position sensors has been documented in works published by different groups, implementations differ depending on intended sensor uses. Sensors based on this principle were developed in the context of microscopy [4,5,12], robotics [8], metrology [6], quality assurance (QA)/quality control (QC) and motion control [10,11,12,13], or general purposes [3,6,9]. 

HORIBA France developed a vision-based 3-DOF encoder [5,9,10,11,12] that was designed to provide motion control to scientific instruments and quality-control solutions for motion systems. It is commercially available under trademark nanoGPS OxyO^®^. While x, y precision and accuracy of the nanoGPS OxyO^®^ sensor were already reported in previous works [11,12], the accuracy of the angular measurement has not yet been thoroughly investigated. This paper aims to (a) determine the precision and accuracy of the angular orientation provided by the encoder; and (b) contribute to a better understanding of angle accuracy of vision-based 3-DOF position sensors, as it is, to our knowledge, the first metrological investigation of such a system in terms of angle accuracy. 

The present understanding of the capabilities of 3-DOF vision-based sensors is limited from a metrology point of view. It was established that this method has subpixel resolution [3,4,6,7,8,9,11]. When expressed in the object plane, reports on precision vary between nm fractions to several tenths of nm. This shows that there is large impact of the design of the system (in particular, the magnification of the imaging optics) on the performance of 3-DOF encoders. This is consistent with the intuition that observing a patterned plate with a microscope yields better precision than when observing a patterned plate with a conventional camera. 

Several papers also provide indications on angle precision. Chen reported 1.2 µrad standard deviation and 2.5 µrad systematic error on a travel of 2 mrad [6], Kim reported a resolution of 0.001° (i.e., 17 µrad) [7], and the Sandoz group reported a precision of 4 µrad [9]. The precision of the nanoGPS OxyO^®^ encoder was determined to be slightly better than 10 µrad [10]. However, to our knowledge, no vision-based 3-DOF sensor has yet been investigated in terms of angle accuracy.

The characterization of angular encoders at the microradian level is a sophisticated metrological problem. The National Metrology Institute of Italy (INRIM) has much experience in high-accuracy angular metrology [14,15,16]. Hence, the nanoGPS Oxyo^®^ system was compared with respect to the INRIM national angle standard, rotating encoder angle comparator (REAC) [16].

## 2. Equipment and Comparison Procedure

### 2.1. NanoGPS Oxyo^®^ Encoder

**Figure 1:**
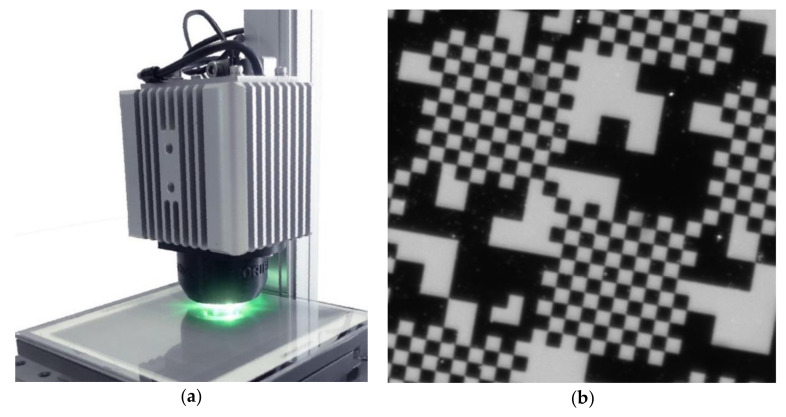
nanoGPS OxyO^®^ encoder. (**a**) photo of whole device including scale and read head; (**b**) detail of scale image taken by read head.

The nanoGPS setup is represented in Figure 1a. It comprised a scale and a read head. The scale exhibited a 2D pattern, as shown on Figure 1b, and the read head included a monochrome camera, an objective, and an illumination system, and provided images of the scale.

The acquired images by the read head were treated via nanoGPS software [5]. The processing software included Fourier analysis and advanced cross-correlation analysis. It provided the position and orientation, along with the magnification between scale and image, expressed in pixel per µm.

The nanoGPS read head used in this study was based on a 1920 × 1920 pixel sensor. It featured darkfield illumination based on green light-emitting diodes (LEDs). Scales comprised metallic patterns deposited on the transparent substrate and white backing that diffused the LED light. We investigated two scales:

- one 125 × 125 mm scale made of fused silica with white backing on the side opposite to the metal patterns, and
- one 25 × 75 mm scale made out of float glass with white backing on the side with metal patterns. For this slide, the scale was read through its substrate.

The nanoGPS software that interpreted the images of the scale into a position was encapsulated as a dynamic-link library. This software was calculation-intensive, and calculation time needed to extract the position information depended on processor performance and image size. While the time required for the processing of a full resolution image is about 1 s with a medium-performance PC, it decreased nearly linearly with the area of the image.

In previously published works, we established:

- precision on the x, y position was about 1 nm rms [12].
- accuracy of the x, y stage, was determined to be better than 100 nm, over 80 mm travel [11]. The quartz scale was used for this investigation. Though 100 nm accuracy over 80 mm travel is already excellent performance that exceeds the requirements of any system, it was not precisely thermally stabilized, and actual performances may be even better, as the limiting factor in this investigation was the control system.
- The solution of the φ_z_ angle was about 10 µrad [10].

The focus of this paper was the characterization of the angular performances of the nanoGPS and the assessment of its angular accuracy.

### 2.2. INRIM National Angle Standard

The INRIM national angle standard, rotating encoder angle comparator (REAC), is based on a high-accuracy angular encoder, ERA 4200, manufactured by Heidenhain, with 40,000 lines and 20 μm pitch. The encoder continuously rotates and is read by two heads, one fixed to the reference frame and the other fixed to the moving table. The phase difference between the two heads’ signals is proportional to the rotation of the moving table. In this configuration, typical encoder errors due to eccentricity and manufacturing are cancelled since phase measurement is averaged over an entire revolution of the encoder. Moreover, the system is free in principle from nonlinearity since phase measurement is performed on a dynamic signal, so it is not influenced by signal interpolation between graduation lines. The accuracy of the system was evaluated at the level of hundreds of nanoradians. A detailed description of the facility can be found in [16].

### 2.3. Experiment-Comparison Details

**Figure 2:**
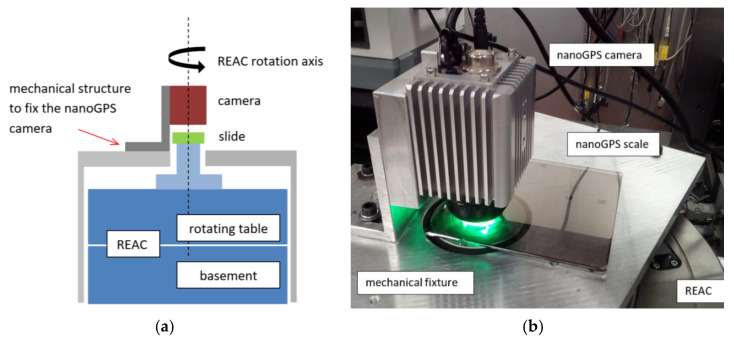
(**a**) Scheme and (**b**) picture of experiment setup for comparison between nanoGPS OxyO^®^ encoder and rotating encoder angle comparator (REAC).

The scheme and picture of the experiment setup for the comparison between nanoGPS OxyO^®^ encoder and REAC are shown in Figure 2. The nanoGPS scale was glued on a mechanical part, in turn fixed to the REAC rotating table, and centered on its rotation axis. The nanoGPS camera was mounted vertically on an L-shaped structure fixed to the reference frame and aimed at the scale. The mounting had some degrees of freedom to allow for the alignment of the camera along the REAC rotation axis. The vertical distance between camera and scale was not a critical issue, since the nanoGPS was equipped with a liquid lens that could adjust the focus of the camera in a range of some millimeters. When the REAC table rotated on an angle α_REAC_, the camera observed the scale rotated by the same angle, and the nanoGPS system measured angle α_OxyO_. Therefore, the comparison between the two instruments simply consisted of the comparison between the two measured angular values, α_OxyO_ and α_REAC_.

The procedure was automated with software implemented in LabVIEW that moved the REAC table and collected the angular values measured by the two systems for a selected sequence of angular intervals. The experiment setup was placed on a granite table in the underground angle-standard laboratory at INRiM.

## 3. Comparison Results and Discussion

### 3.1. Precision and Accuracy of NanoGPS OxyO as an Angle Encoder

**Figure 3:**
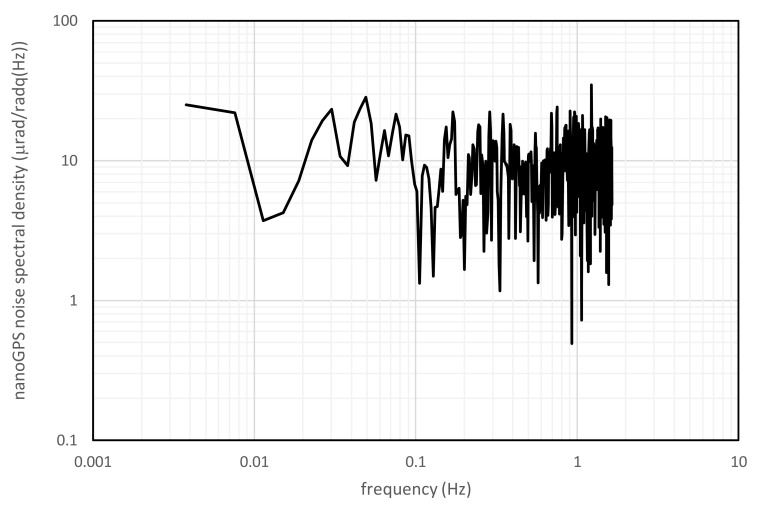
Noise spectral density of nanoGPS OxyO^®^ system for series of 960 x 960 pixel images grabbed and processed at 3 Hz in stationary conditions for 5 min.

In order to evaluate nanoGPS OxyO precision as a function of image size, angular values were measured by the two systems in stationary conditions, i.e., with the REAC table latched. The time needed by the program to process a full resolution (2000 × 2000 pixels) image was non-negligible (about 1 s). In this condition, the noise of the angular measurement was about 5 µrad, calculated as the standard deviation of 50 images. Image-processing time was almost linearly reduced, decreasing image size, but clearly noise was increased, and accuracy was degraded. For example, reducing the size to 480 × 480 pixels, image-processing time was decreased below 0.1 s and noise was increased by a factor of 4 at least, which was consistent with the statistical reduction of the number of pixels by a factor of 16. As an example, Figure 3 shows the noise spectral density of the nanoGPS OxyO^®^ system for 960 × 960 pixel images, grabbed and processed at a frame rate equal to 3 Hz.

On the basis of this consideration, the following analysis was carried on with a 960 × 960 pixel image as a trade-off between resolution and acquisition time.

**Figure 4:**
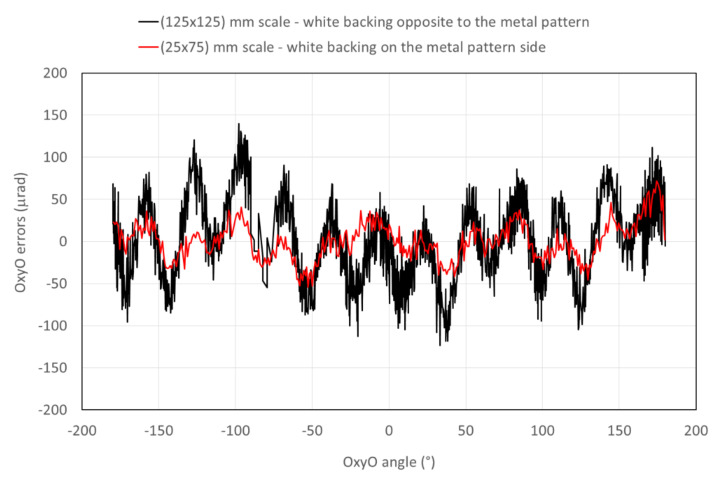
Differences between angular values measured by OxyO system and REAC.

In order to evaluate nanoGPS accuracy, the REAC table was rotated in steps of 5 mrad up to 2π (hence, 1296 points per revolution). Due to the footprint of the 125 × 125 mm scale, it was not possible to explore the entire 2π angle in a unique position of the scale. Hence, four different measurements at the four vertices of the scale were performed, each time exploring an angular interval of about 2π/3. Afterwards, measurements were rearranged and stitched up as a function of the rotation angle. Figure 4 shows differences between angular values measured by the OxyO^®^ system, with the two different scales and the angles measured by REAC plotted against the rotation angle. The black line represents the errors of the OxyO^®^ system obtained with the 125 × 125 mm scale, with white backing on the side opposite to the metal patterns. Errors were encompassed within ±145 µrad, but nonlinearity with a period of about 30° is clearly visible. 

**Figure 5:**
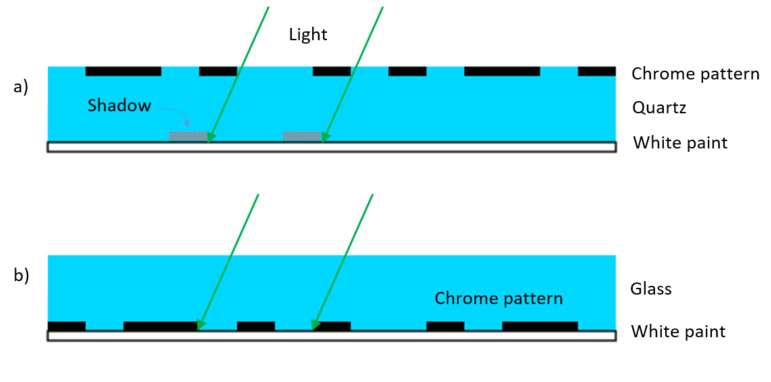
Vertical section sketch of two scales: (**a**) scale made out of quartz with white backing on the side opposite to the metal patterns, and (**b**) scale made out of float glass, with white backing on the side with the metal patterns.

We interpreted this nonlinearity as an effect due to the shadow of the pattern projected by LEDs on the white background. Indeed, the lighting system was made of 3 LEDs at 120°. Instead of performing a complex simulation, in order to confirm this hypothesis, we built a new scale where the white paint was on the patterned side, and the sample was observed through the glass. A vertical section sketch of the two different scales is shown in Figure 5. The shadow effect for the scale with a white diffuser on the side opposite to the metal pattern is clearly visible. 

A new set of measurements was performed with the second scale. An average of 5 sets of measurements is represented by the red curve in Figure 4. The error was indeed reduced by at least a factor of 2 and was of the order of other random errors. Errors were encompassed within ±63 µrad.

### 3.2. Illumination Conditions

The above experiment showed that illumination conditions have an impact on the ultimate angle accuracy that can be achieved. The read head presented here integrated off-angle illumination, and the diffusive surface appeared as white on the image. This illumination mode is also called “dark-field” illumination. A dark field has the advantage of being simpler to implement, but it may also have the disadvantage of being more sensitive to dust and different artefacts; dust particles are clearly seen on Figure 1b. NanoGPS technology proved to be largely immune to a reasonable quantity of dust and scratches, which can be easily understood as most information is retrieved in the Fourier space; nevertheless, it might, to a certain extent, affect ultimate precision and accuracy.

Nano-GPS technology can also be used with straight-through illumination (also called “bright-field” illumination). The authors in [12] reported examples where nanoGPS OxyO scales were read by optical microscopes in bright-field conditions. Bright-field illumination is not affected by shading effects as those reported in Figure 5, and less affected by dust particles.

### 3.3. Discussion: Environmental and System-Design Influence on Measurement Uncertainty 

The nanoGPS OxyO system appears to be a highly performant 3-DOF vision-based absolute encoder. If the literature on vision-based position sensors has solidly established that such systems could achieve subpixel accuracy, their ultimate performance is not well-known. The present study is a contribution to this knowledge. 

Table 1 provides a breakout of different elements of the nanoGPS encoder and of its environment, and considerations about their contribution to measurement uncertainty.

Measurements were performed in optimal conditions. In fact, the angle-standard laboratory was thermally stabilized at 20 ± 0.1 °C. Moreover, systems were placed on a granite table that rests directly on the basement floor of the building, thus reducing the influence of mechanical vibrations. Finally, it was demonstrated that REAC stability was of the order of 0.05 µrad on a time interval of 100 s [16]. Hence, environmental and thermomechanical influences can be considered negligible compared to the performance of the nanoGPS system in the experiment reported here.

Details of the algorithmics and pattern design of the nanoGPS encoder are expected to have an influence on ultimate performance, but these performances can presently be assessed only through experiment evidence. 

Pattern accuracy is, of course, expected to be a key element for the overall uncertainty of the system. The patterned plate used in the nanoGPS OxyO system is produced by equipment dedicated to semiconductors, and it owes its precision to the highly sophisticated and accurate positioning systems used in this industry [2]. Another key factor is that the image sensor also had perfectly regular and orthogonal pixels, also produced by the semiconductor industry. As a consequence, the nanoGPS OxyO system can be viewed as a convenient “solid record” of high-precision positioning machines deployed in the semiconductor industry. This is somewhat similar to gratings employed as linear scales for conventional linear encoders. They are produced by a laser interferometric method, and they are used as solid interferometers, more convenient than the original laser interferometer used to produce them. 

The thermal stability of the patterned plate obviously affects accuracy, but it is also possible to provide a correction on the basis of the coefficient of the plate’s thermal expansion.

A number of other aspects of the system, including image sensor, imaging optics, and the thermal and mechanical properties of the read head can have a slight impact on the measurement uncertainty of the system. A detailed and quantitative estimation of these factors is the focus of future analysis.

## 4. Applications

nanoGPS OxyO encoders are very useful as built-in encoders in complex mechanical systems, as they allow the determination of 3 inplane degrees of freedom of a mechanical system [12,13]. Unlike linear and rotary encoders, their integration should be considered at the systemrather than at the component level.

For systems based on actuators that have built-in encoders, many sources of errors (such as Abbe’s error, mechanical lashes, thermal expansion, and frame deformation) that affect the position of the mechanical part of interest regarding the frame of interest cannot be apprehended by integrated encoders [2]. As a consequence, nanoGPS encoders can be very efficient for calibration and quality-control tasks.

**Figure 6:**
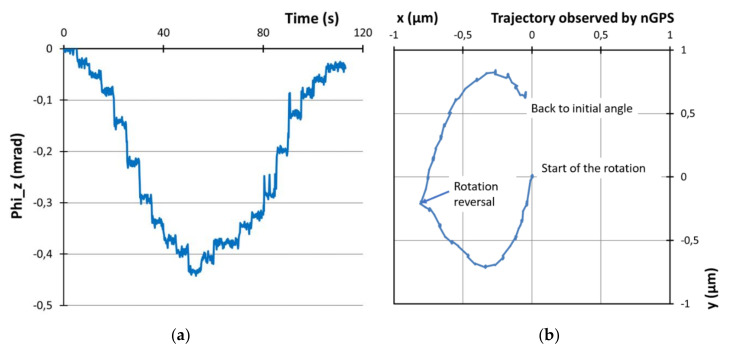
Investigation of motion of fluid bearing using nanoGPS encoder: (**a**) measured angle; (**b**) displacement in the (x, y) plane.

As a simple illustration, we investigated the properties of a rotation bearing with nanoGPS. We placed a 5 × 5 mm^2^ nanoGPS scale on a rotary bearing (fluid bearing taken from a hard-disk drive). We slightly rotated the bearing by pushing a lever connected to the bearing by using a step actuator. Then, we rotated the bearing back toward its initial position. The nanoGPS records are presented in Figure 6. Figure 6a shows that the rotation proceeded through rather irregular steps, up to an angle of 420 µrad, and that it returned to its original orientation within 25 µrad. However, Figure 6b shows that the movement of the rotation bearing was not pure rotation, as the movement of the scale in x, y was not a small circular arc (as would be anticipated in the case of pure rotation), but 2D displacement by more than half a micron with significant hysteresis. This showed that the bearing had travel that could not be approximated as pure rotation; in that case, micrometric accuracy of the positioning system is required. This can be determined in a very simple way with a nanoGPS encoder, while it would probably be much more difficult to implement laser interferometry, which is a well-established technique to measure small displacements, as it requires excellent laser alignment that may not be easy to implement on a rotating system.

## 5. Conclusions

A 3-DOF absolute encoder based on nanoGPS OxyO technology was compared with the national angle standard of INRIM in order to assess its angular performance. The encoder was based on a special patterned scale and a reading head that included a monochrome camera, objective, and LED illumination system. Nonlinearity with a period of about 30° was observed. Error was strongly reduced by manufacturing a new scale allowing for more uniform illumination. Precision better than 5 µrad and accuracy better than 63 µrad over 2π were demonstrated. While the angular precision of vision-based 3-DOF has been reported in the literature, this is the first assessment of angle accuracy performed using a metrological encoder on a system belonging to this class of position-measurement devices. The application of nanoGPS OxyO technology to the characterization of a rotation bearing was illustrated, and it highlights its capability to be simply integrated into a mechanical system to be measured.

## Figures and Tables

**Table 1:** Impact on accuracy of different elements of nanoGPS encoder and its environment.

Influence on Accuracy	Design	Patterned Plate			Read Head					Environment
	Algorithmic & Pattern Design	Pattern Accuracy	Dust and Scratches	Thermal Stability	Image Sensor	Imaging Optics	Thermal Stability	Illumination	Mechanical Stability	Thermal and Mechanical Stability
Present experiment & nanoGPS OxyO system	Proprietary conception	Accuracy better than 100 nm over 125 mm @21,6°C	Performance unaffected by a limited number of defects	Coefficient of termal expansion is low (0,5E-6/K) for Quartz	Image size 5 times larger than minimum	Proprietary design	Read head includes temperature sensor	Shading by patterns impacts accuracy	Proprietary design	Superior environment of INRiM laboratory
Remarks	Up and down scalable	Determined by pattern fabrication	Absolute encoder: recovers from image loss	Slow variations; temperature correction can be calculated	Nr of image pixel can be adjusted experimentally	Scalable; low distorsions at the center of field	Operate after Read Head temperature stabilization	Dark Field, Bright Field, or Transmission	Can be determined using nanoGPS	The nanoGPS system can be used to investigate the environment stablity
